# Determinants of pre-eclampsia among women attending delivery services in public health institutions of Debre Tabor Town: a case–control study

**DOI:** 10.1186/s12978-022-01463-1

**Published:** 2022-07-08

**Authors:** Alemu Degu Ayele, Zemenu Alemu Tilahun

**Affiliations:** Department of Midwifery, College of Health Sciences, Debre Tabor University, Debre Tabor, Ethiopia

**Keywords:** Determinant, Ethiopia, Pre-eclampsia

## Abstract

**Background:**

The burden of pre-eclampsia has been continued as a public health importance in both developed and developing countries. However, the consequence of the disease is significantly high in developing countries, where treatment may be unsuccessful due to unclear etiology and late presentation of cases. The determinants of pre-eclampsia are not well known in the Ethiopian population specifically in the study area. Therefore, this study was aimed to identify the determinant factors of pre-eclampsia among women attending delivery services in public health institutions of Debre Tabor Town.

**Methods:**

Unmatched case–control study was conducted from December 1, 2020, to January 15, 2021, among 264 mothers (88 cases and 176 controls). A case–control incidence density sampling technique was applied and data were collected using an interviewer-administered pre-tested questionnaire. Data were entered using Epi-data version 4.2 and analyzed by statistical package for social science (SPSS) version 23. Bivariate and multivariable logistic regression analyses were conducted. Finally, a significant association was declared at a p-value of ≤ 0.05 with 95% CI.

**Results:**

Young age at menarche (10–15 years) (AOR: 7.69; 95% CI: 3.10–25.29), status of the current pregnancy (AOR: 5.88; 95% CI:2.93–22.42), new partner (AOR: 4.16; 95% CI: 3.49–17.03), family history of pre-eclampsia (AOR: 1.52; 95% CI: 1.40–4.18), and alcohol drinking (AOR: 2.18; 95% CI: 2.04–11.79) were found to be significantly associated with pre-eclampsia.

**Conclusions:**

The current study identified that young age at menarche, the status of current pregnancy, new partner, family history of pre-eclampsia, and alcohol drinking were some of the determinant factors of pre-eclampsia. We suggested that health care providers should use these identified factors as a screening means for prediction, early diagnoses, and timely interventions of pre-eclampsia. Health care professionals should also deliver information regarding the risk of alcohol drinking during pregnancy in the preconception period and at early antenatal care services.

## Plain language summary

Pre-eclampsia has continued as a burden in both developed and developing countries. However, the consequence of the disease is significantly high in developing countries, where treatment may be unsuccessful due to unclear etiology and late presentation of cases.

Unmatched case–control study was conducted from December 1, 2020, to January 15, 2021, among 264 mothers (88 cases and 176 controls). A case–control incidence density sampling approach was applied and data were collected using an interviewer-administered pre-tested questionnaire.

Young age at menarche (10–15 years), the status of the current pregnancy, new partner, family history of pre-eclampsia, and alcohol drinking were found to be the significant determinant factors of pre-eclampsia. The authors recommended that health care providers should use the identified factors as a screening means for prediction, early diagnoses, and timely interventions of pre-eclampsia. Health care professionals should also deliver information regarding the risk of alcohol drinking during pregnancy in the preconception period and at early antennal care service.

## Introduction

Pre-eclampsia (PE) is a pregnancy-related hypertensive disorder characterized by a systolic blood pressure of ≥ 140 mmHg and a diastolic blood pressure of at least 90 mmHg in two or more consecutive occurrences of at least four hours apart or a diastolic blood pressure of at least 110 mmHg on any one occurrence, plus proteinuria of at least 300 mg per 24 h or ≥ + 2 on a urine dipstick after the 20th week of gestation in a previously normotensive and non-proteinuric pregnant woman (1, 2).

PE has continued as a burden in both developed and developing countries (3, 4). However, the consequence of the disease is significantly high in developing countries, where treatment may be unsuccessful due to its unclear etiology and late presentation of cases. Even though it is difficult to prevent the condition of pre-eclampsia, early identification and proper management of women with pre-eclampsia can minimize its catastrophic effect and long-term sequel (3, 5).

Globally pre-eclampsia affects 5–10% of all pregnancies (6). However, in developing countries, the incidence reaches up to 16.7% and it is responsible for 40% to 60% of maternal deaths (6, 7). According to the world health organization (WHO) estimation, the incidence of pre-eclampsia is seven times higher in developing countries (2.8% of live births) than in developed countries (0.4%) (8).

In Ethiopia PE is one of the major five leading causes of maternal death (hemorrhage, obstructed labor, pre-eclampsia/eclampsia, unsafe abortion, sepsis) and it accounts for 11% of maternal death from these major causes (9).

Even though the government of Ethiopia is worked hard to improve the quality of healthcare services through continuous capacity-building (ensuring pre-service and in-service training for health providers to detect and manage PE), logistics (making supplies available to health institutions for the management of PE), and supportive supervision and mentoring, the late antenatal care initiation and insufficient evidence on causes and pathophysiology of pre-eclampsia remain a challenge to control it. As a result, the burden of the PE remains as a public health problem that can progress to catastrophic events including maternal death (10).

Studies showed that various risk factors have been associated with pre-eclampsia/ eclampsia (11–14). However, the full range of determinant factors affecting pre-eclampsia has not been well known in the Ethiopian population specifically in the study area. Therefore, this study was aimed to identify some determinant factors of pre-eclampsia with a view of informing policy, creating awareness, and formulating strategies to improve antenatal care and delivery services among women attending delivery services in public health institutions of Debre Tabor Town Northwest Ethiopia.

## Methods

### Study design and setting

A facility-based unmatched case–control study was conducted in public health institutions of Debre Tabor Town from December 1, 2020, to January 15, 2021. Debre Tabor Town is the capital city of the south Gondar zone Amhara regional state which is located 665 km far from Addis Ababa (the capital city of Ethiopia) in a northwest direction and 103 km from Bahir Dar. The town has four public health institutions (one comprehensive specialized hospital and three health centres) that include Debre Tabor Comprehensive Specialized Hospital (DTCSH), Leul Alemayehu Health Centre (LAHC), Gaffat Health Centre (GHC), and Debre Tabor Health Centre (DTHC). These institutions provide community health care including maternal and child health services. All pregnant women attending delivery services in public health institutions were the source population. While women attending delivery services in public health institutions during the study periods were the study population.

### Inclusion criteria

The cases were women with systolic blood pressure ≥ 140 mmHg or diastolic blood pressure ≥ 90 mmHg on two separate readings taken at least four hours apart or diastolic blood pressure of at least 110 mmHg on any one occurrence, plus proteinuria of at least 300 mg per 24 h or ≥ + 2 on a urine dipstick after the 20^th^ week of gestation in a previously normotensive and non-proteinuric pregnant woman and women who have given written informed consent to participate in the study and attending delivery service in the hospital. The diagnosis involves history taking, physical examination, and laboratory investigation results. Women’s charts were reviewed to support the diagnosis.

Controls were women who were attending delivery services in the same health institutions during the same time and were not diagnosed as pre-eclamptics/ eclamptics. All cases and controls were seen by the most senior health professionals (obstetricians, general practitioners, and or midwives) in each health institution.

### Exclusion criteria

Women with known chronic hypertension disorders and renal disease and attending delivery services in the public health institutions were excluded from the study.

### Sample size determination and sampling procedures

The sample size was determined by double population proportion formula using EPI-INFO version 7 software by considering 95% CI, 80% power, 5% margin of error, and 1:2 cases to control ratio. The different sample size was calculated by using different risk factors of pre-eclampsia, but the maximum sample size was gained by taking primigravida as a determinant factor for PE (15) where the proportion of PE among cases and controls were 60% and 40% respectively. Therefore, the calculated sample size was 240 (80 cases and 160 controls). The final sample size after considering a 10% non-response rate was 264 (88 cases and 176 controls).

All women admitted for labour and delivery services who fulfilled the inclusion criteria in the health institutions were consecutively involved until the desired sample size was attained. Case–control incidence density sampling technique was applied to select the cases consecutively as they are diagnosed to have pre-eclampsia/eclampsia until the calculated sample size was attained. For every case included, two control mothers who came for delivery services in the same facility and same day/week were identified by using a simple random sampling technique. The sample size was distributed for each health institution proportionally based on their delivery case flow. According to information got from each institution the monthly average delivery report was (330 for DTSH, 125 DTHC, 105 GHC, and 100 LAHC). The total monthly delivery report was 660. Therefore, the allocated samples for each institution were 132 (44 cases, 88 controls) for DTSCH, 50 (17 cases, 33 controls) for DTHC, 42 (14 cases, 28 controls) for GHC, and 40 (13 cases 27 controls) for LAHC.

### Data collection tools and procedure

Data were collected through a face-to-face interviewer-administered technique using a structured and pre-tested questionnaire. Mothers’ chart reviews were also performed to abstract clinical laboratory findings such as protein urea, organ function test (creatinine, liver enzymes), and findings of HELLP (Hemolysis, Elevated liver enzyme, Low platelet counts) syndrome. The tool was first prepared in English and translated to the local Amharic language for simplicity and then back to English to assure consistency by two language experts. The questionnaire has four parts that include socio-demographic characteristics like (age, educational status, marital status, residency, occupation) obstetrical and reproductive health characteristics like (gravidity, age at menarche, age at first pregnancy, ANC follow-up, the status of pregnancy, family history of PE), medical and lifestyle related characteristics like (family history of DM, family history of hypertension, physical exercise, drinking habit, and smoking habit). Four diploma midwives were hired for data collection and supervised by two BSc midwives.

### Data quality control

To maintain the quality of the data, the tool was prepared after an intensive review of relevant literature searches. A pre-test was done on 5% (13 mothers) in Debre Tabor Hospital before the actual data collection period and modifications such as wording errors, skip patterns, and jargon words were made. Besides these, data collectors and supervisors were trained for two days on the aim of the study, items of the questionnaire, confidentiality, and the data collection process. Finally, the collected data were cleaned and compiled daily by supervisors and principal investigator.

### Analysis and interpretation

The collected data were cleaned, coded, and entered using Epi-data software version 4.2, and analyzed by statistical package for social science (SPSS) version 23. Descriptive statistics was used to describe the study populations using measures of frequency and central tendency and summarized using tables compared between cases and controls.

Bivariate and multivariable logistic regression analyses were performed. In bivariate analysis variables that have an association with the development of PE with a p-value of less than 0.2 were fitted into a multivariable logistic regression model for further analysis. Finally, variables that have a significant association with PE at a p-value of 0.05 with 95% CI and AOR was declared as a statistically significant variable.

### Ethical consideration

Ethical clearance was sought from Debre Tabor University College of Health Sciences research review committee. Also, a support letter was granted to each health institution. Moreover, all the study participants were informed about the purpose of the study, their right to refuse, and assurance of confidentiality. Then, written consent was obtained from each study participant. Strict confidentiality was assured through anonymous of all recording and coding of the questionnaire. This study was following the Declaration principles of Helsinki.

## Results

### Socio-demographic characteristics

A total of 264 (88 cases and 176 controls) labouring mothers were enrolled with a response rate of 100%. This was a multicentral study conducted in Debre Tabor Comprehensive Specialized Hospital which has 132 participants, Debre Tabor Health Centre with 50 participants, Gaffat Health Centre with 42 respondents, and Leul Alemayehu Health Centre with 40 respondents. The mean age of the study participant was 27.67 (SD ± 6.42) years; with the mean maternal age of cases being 25.26 (SD ± 5.45) and controls 28.88 (SD ± 6.55) years. Forty-three (48.9%) of cases and eighty-six (48.9%) of controls were found with the age group of 25–34 years. Seventy (79.5%) of the cases and one hundred and seventy (96.6%) of the controls were Amhara by their ethnicity. The majority of the study participants were orthodox Christian followers with fifty-four (61.4%) among cases and one hundred and sixty one (91.5%) among controls (Table 1).

**Table 1:** Socio-demographic characteristics study participants in public health institutions of Debre Tabor Town, Northwest Ethiopia, December 1, 2020 to January 15, 2021(N = 264)

Characteristics	Pre-eclampsia	
	Case (88) No (%)	Control (176) No (%)
Age		
15–24	35 (39.8)	41 (23.3)
25–34	43 (48.9)	86 (48.9)
35–49	10 (11.3)	49 (27.8)
Mean (SD), year	25.26 (5.45)	28.88 (6.55)
Residency		
Urban	36 (40.9)	85 (48.3)
Rural	52 (59.1)	91 (51.7)
Ethnicity		
Amhara	70 (79.5)	170 (96.6)
Others*	18 (20.5)	6 (3.4)
Religion		
Orthodox	54 (61.4)	161 (91.5)
Muslim	13 (14.8)	12 (6.8)
Others**	21 (23.8)	3 (1.7)
Educational status		
No formal education	24 (27.3)	41 (23.3)
Primary	8 (9.1)	41 (23.3)
Secondary	43 (48.9)	73 (41.5)
Tertiary	13 (14.7)	21 (11.9)
Occupational status		
House wife	43 (48.9)	94 (53.4)
Government employee	17 (19.3)	44 (25.0)
Private business	23 (26.1)	31 (17.6)
Others***	5 (5.7)	7 (4.0)
Marital status		
Married	78 (88.6)	139 (79.0)
Others****	10 (11.4)	37 (21.0)

*Oromo, tigray, gurage ** protestant, catholic *** employed at private sector, student and job finder **** single, widowed, separated

### Obstetrics and reproductive health characteristics of the study participants

Among the total participants, 29 (33.0%) of cases and 163 (92.6%) of controls were multigravidas. Concerning antenatal care (ANC) follow-up, 80 (70.4%) and 150 (85.2%) of mothers had ANC follow-up among cases and controls respectively. More than half of the cases 49 (55.7%) had a family history of PE among cases, whereas only 12 (6.8%) of controls had a family history of PE. Regarding the status of the current pregnancy, about 46 (52.3%) among cases and 12 (6.8%) among controls were unwanted and unplanned (Table 2).

**Table 2:** Obstetric and reproductive health characteristics of study participants in public health institutions of Debre Tabor Town, Northwest Ethiopia, December 1, 2020 to January 15, 2021(N = 264)

Characteristics	Pre-eclampsia	
	Case (88)	Control (176)
Gravidity		
Primi	59(67.0)	13(7.4)
Multi	29(33.0)	163(92.6)
Age at menarche		
≤ 15 years	30 (34.1)	13 (7.4)
> 15 years	58 (65.9)	163 (92.6)
Age at first pregnancy		
≤ 20 years	52 (59.1)	81 (46.0)
> 20 years	36 (40.9)	95 (54.0)
ANC follow up		
Yes	80 (70.4)	150 (85.2)
No	8 (29.6)	26 (14.8)
Change partner		
Yes	43 (48.9)	19 (10.8)
No	45 (51.1)	157 (89.2)
History of abortion		
Yes	6 (6.8)	26 (14.8)
No	82 (93.2)	150 (85.2)
Family history of PE		
Yes	49 (55.7)	12 (6.8)
No	39 (44.3)	164 (93.2)
Condition of pregnancy		
Wanted	28 (31.8)	142 (80.7)
Unwanted	46 (52.3)	12 (6.8)
Mistimed	14 (15.9)	22 (12.5)

*ANC* antenatal care, *PE* preeclampsia

### Medical and life style related characteristics

The proportion of history of diabetes mellitus was 27 (30.7) in the cases versus 10 (5.7%) in the control groups. Meanwhile, 21 (23.9%) of participants among cases had a family history of hypertension while 17 (9.7%) of participants among controls had family history of hypertension. About 55 (62.5) mothers from the cases and 71 (40.3%) from the controls drink alcohol at least three times per week (Table 3).

**Table 3:** Medical and Life style related characteristics of the study participants in public health institutions of Debre Tabor Town, Northwest Ethiopia, December 1, 2020 to January 15, 2021(N = 264)

Characteristics	Pre-eclampsia	
	Case (88)	Control (176)
Family history of DM	27 (30.7)	10 (5.7)
Family history of HTN	21 (23.9)	17 (9.7)
History of paternal hypertension	15 (17.0)	9 (4.1)
Smoking habit	16 (18.2)	21 (11.9)
Alcohol intake	55 (62.5)	71 (40.3)
Coffee intake	66 (73.9)	106 (60.2)
Physical exercise	40 (45.5)	78 (44.3)
Vegetable’s intake	77 (87.5)	144 (81.8)
Fruit intake	75 (85.2)	159 (90.3)

*DM* diabetes mellitus, *HTN* hypertension

### Determinants of pre-eclampsia among the study participants

According to the bivariate analysis finding, maternal age (≥ 35 years), status of the current pregnancy, age at menarche, new partner, family history of PE, family history of gestational diabetic mellitus, and alcohol intake at least three times per week were significantly associated with the development of pre-eclampsia. After adjusting the possible confounding factors, the multivariable logistic regression analysis showed that status of the current pregnancy, age at menarche, new partner, family history of PE, and alcohol intake of at least three times per week were found to be significantly associated with pre-eclampsia.

Young age at menarche (10–15 years) was nearly eight times more likely to suffer from pre-eclampsia (AOR: 7.69; 95% CI: 3.10–25.29) than mothers whose age at menarche were greater than 15 years. Compared with those women whose pregnancies were wanted and planned, women with unwanted and unplanned pregnancies were nearly six times at greater risk of developing pre-eclampsia (AOR: 5.88; 95% CI: 2.93–22.42). The odds of developing pre-eclampsia was four times higher for those women who had a new partners (AOR: 4.16; 95% CI: 3.49–17.03) as compared to their counterparts.

Moreover, mothers who had a family history of pre-eclampsia were nearly two times more likely to suffer from pre-eclampsia (AOR: 1.52; 95% CI: 1.40–4.18) as compared to those mothers who had no family history of PE.

Once more, mothers who drink alcohol at least three times per week during pregnancy were two times more likely to develop pre-eclampsia (AOR: 2.18; 95% CI: 2.04–11.79) as compared to those mothers who did not take alcohol during pregnancy (Table 4).

**Table 4:** Bivariable and multivariable analysis of determinants of pre-eclampsia among mothers attending delivery in public health institutions of Debre Tabor Town, Northwest Ethiopia, December 1, 2020 to January 15, 2021 (N = 264)

Variables	Pre-eclampsia		COR (95%CI)	AOR (95%CI)	*P-value*
	Case (%)	Control (%)			
Age in years					
15–24	35 (39.8)	41 (23.3)	1	1	
25–34	43 (48.9)	86 (48.9)	1.707 (0.955–3.052)	0.477 (0.140–1.626)	0.237
≥ 35	10 (11.3)	49 (27.8)	4.183 (1.850–9.460)	2.436 (0.545–10.890)	0.244
Educational status					
No formal education	24 (27.3)	41 (23.3)	1.058 (0.449–2.488)	1.511 (0.207–11.048)	0.550
Primary	8 (9.1)	41 (23.3)	3.173 (1.137–8.850)	2.031 (0.295–11.989)	0.193
Secondary	43 (48.9)	73 (41.5)	1.051 (0.478–2.310)	1.854 (0.273–12.609)	0.725
Tertiary	13 (14.7)	21 (11.9)	1	1	
Age at menarche					
10–15 years	30 (34.1)	13 (7.4)	6.485 (3.168–13.277)	7.696 (3.105–25.293)	0.001*
15–20 years	58 (65.9)	163 (92.6)	1	1	
Gravidity					
Primi	59 (67.0)	13 (7.4)	1.694 (0.406–1.186)	2.041 (0.408–10.212)	0.385
Multi	29 (33.0)	163 (92.6)	1	1	
Status of pregnancy					
Wanted	28 (31.8)	142 (80.7)	1	1	
Unwanted	46 (52.3)	12 (6.8)	0.051 (0.024–0.109)	5.889 (2.933–22.427)	0.001*
Mistimed	14 (15.9)	22 (12.5)	0.310 (0.142–0.678)	0.095 (0.022–3.402)	0.476
Change partner					
Yes	43 (48.9)	19 (10.8)	0.127 (0.067–0.239)	4.164 (3.494–17.033)	0.035*
No	45 (51.1)	157 (89.2)	1	1	
Family history of PE					
Yes	49 (55.7)	12 (6.8)	0.058 (0.028–0.120)	1.521 (1.402–4.182)	0.001*
No	39 (44.3)	164 (93.2)	1	1	
Smoking habit					
Yes	16 (18.2)	21 (11.9)	1.640 (0.808–3.329)	4.656 (0.998–11.440)	0.055
No	72 (81.9)	155 (88.1)	1	1	
Alcohol intake					
Yes	55 (62.5)	71 (40.3)	1.406(0.240–0.687)	2.187 (2.044–11.797)	0.023*
No	33 (37.5)	105 (59.7)	1	1	

*p-value < 0.05 considered as statistically significant, *PE* pre-eclampsia

## Discussion

In this study, we carried out institutional-based unmatched case–control to determine the socio-demographic, obstetrical, medical condition, and lifestyle (behavioral) factors that affect the development of pre-eclampsia.

According to this study finding, young age at menarche (10–15 years) was significantly associated with pre-eclampsia. The finding was consistent with studies conducted in India (16, 17). The likely explanation might be early menarche (10–15 years) had been associated with amplified risk of developing cardiovascular events (18, 19). Additionally, early menarche is at risk of excessive accumulation of fatty tissue that may lead to obesity which prompts to pre-eclampsia (20).

Our study finding also noted that the status of current pregnancy was significantly associated with the development of pre-eclampsia. Women whose pregnancies were unwanted and unplanned were more likely to suffer from pre-eclampsia than their counterparts. To the best of our knowledge, this is the new finding that identified the status of pregnancy as a determinant factor of pre-eclampsia. The possible explanation might be mothers with an unwanted and unplanned pregnancy may have no healthcare-seeking behavior, including ANC follow-up which helps the mother to get awareness and counseling regarding danger signs of pregnancy mostly happened as a result of pre-eclampsia like headache, blurring of vision, epigastric pain, and its possible preventive approach by the health care providers. In the meanwhile, mothers with unwanted and unplanned pregnancies may also have psychological effects by creating stressful life events which is one of the predisposing factors of pre-eclampsia (21).

Moreover, those mothers who had a new partner had an increased risk of pre-eclampsia. This is also the new variable mentioned as a determinant factor for the development of pre-eclampsia. This might be changing parents were focused on the hypothesis that fresh antigens from new parents were a risk factor for PE due to maladaptation.

As mentioned elsewhere, mothers who had a family history of pre-eclampsia had an increased risk of developing pre-eclampsia (16, 22, 23). This result is also noticed in the present study finding. The probable explanation may be due to the acquisition of the risk factors of pre-eclampsia from the partner through hereditary factors. Besides, there may be a possibility of some cardiovascular disorders with a genetic inheritance that increases the likely hood of being pre-eclamptic.

Lastly, the finding of our study noticed that alcohol intake at least three times per week during pregnancy was positively associated with the development of pre-eclampsia. Different studies also mentioned that alcohol consumption during pregnancy was the single most determinant factor for pre-eclampsia (15, 24–27). This might be due to the effect of alcohol consumption on renal function and systemic blood vessels which may expose the mother to hypertension disorder and then this hypertension disorder ultimately leads to pre-eclampsia.

### Strength

The study tried to incorporate more than one institution; the controls were selected from the same institution where the cases were derived on the same day. Besides, it tried to identify new risk factors like the status of pregnancy and new partner as a determinant factor for the first time.

### Limitations

Our study did not end up without limitations. First, there might be a misdiagnosis of mothers due to different parameters like the progress of the condition, availability/quality of laboratory machine, and quality of the professionals. Second, since the data were collected during delivery we might miss some mothers that may develop pre-eclampsia in the postpartum period. Thirdly, since the data were collected during labour and delivery there might be recall bias due to labour pain.

## Conclusion

The current study identified that young age at menarche (10–15 years), the status of the current pregnancy, new partner, family history of pre-eclampsia, and alcohol intake at least three times per week were significantly associated with pre-eclampsia. We suggested that health care providers should use the identified factors as a screening mean for prediction, early diagnoses as well as timely interventions of pre-eclampsia. Health care professionals should also deliver information to avoid drinking alcohol during pregnancy preconceptionally and at early antenatal care services.

## Data Availability

The data that support the review findings of this study are available upon a reasonable request to the corresponding author.

## Abbreviations

ANC: Antenatal care
PE: Preeclampsia

## References

[1] For the Revision IA. A conceptual framework for the revision of the ICD-10 classification of mental and behavioural disorders. World Psychiatry. 2011;10(2):86–92. 10.1002/j.2051-5545.2011.tb00022.x. 10.1002/j.2051-5545.2011.tb00022.x PMC3104876 21633677

[2] American College of Obstetricians and Gynecologists (2013). Hypertension in pregnancy, task force on hypertension in pregnancy. Obstet Gynecol.

[3] Surapaneni T, Bada VP, Nirmalan CP (2013). Risk for recurrence of pre-eclampsia in the subsequent pregnancy. J Clin Diagn Res JCDR..

[4] Report of the National High Blood Pressure Education Program Working Group on High Blood Pressure in Pregnancy. Am J Obstet Gynecol. 2000;183(1):S1-S22. 10.1067/mob.2000.107928. 10920346

[5] Manandhar BL, Chongstuvivatwong V, Geater A (2014). Antenatal care and severe pre-eclampsia in Kathmandu valley. J Chitwan Med College.

[6] Osungbade KO, Ige OK (2011). Public health perspectives of preeclampsia in developing countries: implication for health system strengthening. J Pregnancy.

[7] Martin JN, MY O, Keise SD (2012). Standardized Mississippi protocol treatment of 190 patients with HELLP syndrome: slowing disease progression and preventing new major maternal morbidity. Hypertens Pregnancy.

[8] Lill T, Per M, Rolv S, Camilla S (2008). Previous abortions and risk of preeclampsia. Int J Epidemiol.

[9] Federal Democratic Republic of Ethiopia Ministry of Health. Maternal death surveillance and response (MDSR) technical guideline. Addis Ababa: Federal Democratic Republic of Ethiopia Ministry of Health; 2013.

[10] Maternal and Child Health Integrated Program (MCHIP). Interventions for impact in essential obstetric and newborn care. Addis Ababa: Maternal and Child Health Integrated Program (MCHIP); 2011.

[11] Kahnamouei-aghdam F, Amani F, Hamidimoghaddam S (2015). Prevalence of preeclampsia and eclampsia risk factors among pregnant women, 2011–2013. Int J Adv Med.

[12] Zhang F, Dong L, Zhang C (2011). Increasing prevalence of gestational diabetes mellitus in Chinese women from 1999 to 2008. Diab Med.

[13] Lee CJ, Hsieh TT, Chiu TH, Chen KC, Lo LM, Hung TH (2000). Risk factors for pre-eclampsia in an Asian population. Int J Gynecol Obstet.

[14] Organization WH. Trends in Maternal Mortality: 1990 to 2008. Geneva: World Health Organization; 2010. Available at https://apps.who.int/iris/bitstream/handle/10665/44423/9789241500265_eng.pdf.

[15] Grum T, Seifu A, Abay M, Angesom T, Tsegay L (2017). Determinants of pre-eclampsia/Eclampsia among women attending delivery Services in Selected Public Hospitals of Addis Ababa, Ethiopia: a case control study. BMC Pregnancy Childbirth.

[16] Verma MK, Kapoor P, Yadav R, Manohar RK (2017). Risk factor assessment for preeclampsia: a case control study. Int J Med Public Health.

[17] Ramesh K, Gandhi S, Rao V (2014). Socio-demographic and other risk factors of preeclampsia at a tertiary care hospital, Karnataka: case control study. J Clin Diagn Res JCDR..

[18] Prentice P, Viner RM (2013). Pubertal timing and adult obesity and cardiometabolic risk in women and men: a systematic review and meta-analysis. Int J Obes.

[19] Day FR (2015). Puberty timing associated with diabetes, cardiovascular disease and also diverse health outcomes in men and women: the UK Biobank study. Sci Rep.

[20] Latha K, Judie A (2014). Unveiling the precedence of risk factors for pre-eclampsia: for suitable surveillance and prophylaxis. IJSR Int J Sci Res.

[21] Geller PA (2004). Pregnancy as a stressful life event. CNS Spectr.

[22] Tessema GA, Tekeste A, Ayele TA (2015). Preeclampsia and associated factors among pregnant women attending antenatal care in Dessie referral hospital, Northeast Ethiopia: a hospital-based study. BMC Pregnancy Childbirth.

[23] Mareg M (2020). Determinants of Preeclampsia Among Pregnant Mothers Attending Antenatal Care (ANC) and Delivery Service in Gedeo Zone, Southern Ethiopia: Case Control-Study; Dovepress. Int J Women’s Health.

[24] Lecarpentier E, Tsatsaris V, Goffinet F, Cabrol D, Sibai B (2013). Risk factors of superimposed preeclampsia in women with essential chronic hypertension treated before pregnancy. PLoS ONE.

[25] Shegaze M, Markos Y, Estifaons W, Taye I, Gemeda E (2016). Magnitude and associated factors of preeclampsia among pregnant women who attend antenatal care service in public health institutions in Arba Minch Town, Southern Ethiopia, 2016. Gynecol Obstet (Sunnyvale).

[26] Ye C, Ruan Y, Zou L, Li G, Li C, Chen Y (2014). The 2011 survey on hypertensive disorders of pregnancy (HDP) in China: prevalence, risk factors, complications, pregnancy and perinatal outcomes. PLoS ONE.

[27] Iwama N, Metoki H, Nishigori H, Mizuno S, Takahashi F, Tanaka K (2019). Association between alcohol consumption during pregnancy and hypertensive disorders of pregnancy in Japan: the Japan Environment and Children’s Study. Hypertens Res.

